# Oil Adulteration Identification by Hyperspectral Imaging Using QHM and ICA

**DOI:** 10.1371/journal.pone.0146547

**Published:** 2016-01-28

**Authors:** Zhongzhi Han, Jianhua Wan, Limiao Deng, Kangwei Liu

**Affiliations:** 1 Information College, Qingdao Agricultural University, Qingdao, China; 2 School of Geosciences, China University of Petroleum Huadong, Qingdao, China; Monash University, AUSTRALIA

## Abstract

To investigate the feasibility of identification of qualified and adulterated oil product using hyperspectral imaging(HIS) technique, a novel feature set based on quantized histogram matrix (QHM) and feature selection method using improved kernel independent component analysis (iKICA) is proposed for HSI. We use UV and Halogen excitations in this study. Region of interest(ROI) of hyperspectral images of 256 oil samples from four varieties are obtained within the spectral region of 400–720nm. Radiation indexes extracted from each ROI are used as feature vectors. These indexes are individual band radiation index (RI), difference of consecutive spectral band radiation index (DRI), ratio of consecutive spectral band radiation index (RRI) and normalized DRI (NDRI). Another set of features called quantized histogram matrix (QHM) are extracted by applying quantization on the image histogram from these features. Based on these feature sets, improved kernel independent component analysis (iKICA) is used to select significant features. For comparison, algorithms such as plus L reduce R (plusLrR), Fisher, multidimensional scaling (MDS), independent component analysis (ICA), and principle component analysis (PCA) are also used to select the most significant wavelengths or features. Support vector machine (SVM) is used as the classifier. Experimental results show that the proposed methods are able to obtain robust and better classification performance with fewer number of spectral bands and simplify the design of computer vision systems.

## Introduction

With the development of the society and economy, oil products are becoming more and more important for automobile industry. Driven by the great economic benefit, some unscrupulous traders sold low-value or adulterated oil products instead of high-value oil products in recent years. Many oil refinery factories in China are producing adulterated oil to make more profits according to a report by China Central Television (CCTV) in its annual 3.15 Gala program[1]. They use 90# gasoline, naphtha, aromatics and other additives to produce 93# blend oil. Adulterated oil has not only damaged the consumers’ benefits, but also threated people’s safety. Therefore, to guarantee and promote oil products’ quality, the identification of the qualified oil products and adulterated oil products is extremely essential.

High Performance Liquid Chromatography (HPLC) and Mass Spectroscopy (MS) are well known chemical detection methods, and HPLC has advantages in terms of accuracy and sensitivity [2].Although the result achieved by HPLC is accurate, it is time consuming, inefficient and destructive, and also requires highly trained and qualified professionals. Moreover, the identification cannot be used on-line in the industrial field. Thus, an effective method based on spectral technique and pattern classification technique has been proposed for the identification of the qualified oil products and adulteration products. Because it’s faster, cheaper and nondestructive, it is considered as an alternative method for oil detection. Kim et al were the first to use real-time classification method for petroleum products detection and studied oil products classification of six varieties using near-infrared spectra [3].

However there is limited research on the identification of the oil adulteration, especially using hyperspectral imaging technique. Owing to its advantages, hyperspectral imaging (HSI) which integrates imaging and spectral technique together has been studied extensively in many areas. By analyzing sesame oil, Xie et alachieved 95.59% and 98.53% classification performance by SPA-LS-SVM and CARS-LDA using near-infrared hyperspectral imaging[4].

As pointed out by Kesslerin *Science* [5], oil products samples exhibit bright fluorescence under 365nm ultraviolet light. Actually the mechanism of the phenomenon is much more complicated. Different components and percentage of oil produce different fluorescence. If oil products are adulterated, the color and luminous intensity of fluorescence will be changed. It can be shown in the hyperspectral imaging. Yi et alused wavelet of three-dimensional fluorescence spectrum to classify six oil varieties of four classes under halogen illumination[6].

Besides halogen illumination, UV illumination is also a possible excitation way. Atas et al achieved 90% classification rate of examining aflatoxin-infected chili pepper under UV fluorescence using a hyperspectral imaging system[7].

This paper aims to find a way to identify the oil products and adulterated ones using HSI technique under compound light, halogen illumination and UV excitation. Four radiation indexes which are extracted from each ROI are used as feature vectors. And then a novel feature set of quantized histogram matrix (QHM) and a novel feature selection method based on improved kernel independent component analysis (iKICA) are proposed. The objectives of this work are: 1. to select effective features using feature selection method by our constructed model; 2 to compare the performance of different feature selection models under different light illumination; 3. to find out the quantitative relationships between the spectral information and the oil adulteration. In the following section, we will describe hyper spectral data capture and preprocessing. And then, the feature extraction and selection methods will be introduced in Section 3. Next, we will present and discuss our experimental results in Section 4. Finally, conclusions will be given in Section 5.

## Materials

### Flow of the study

**Fig 1:**
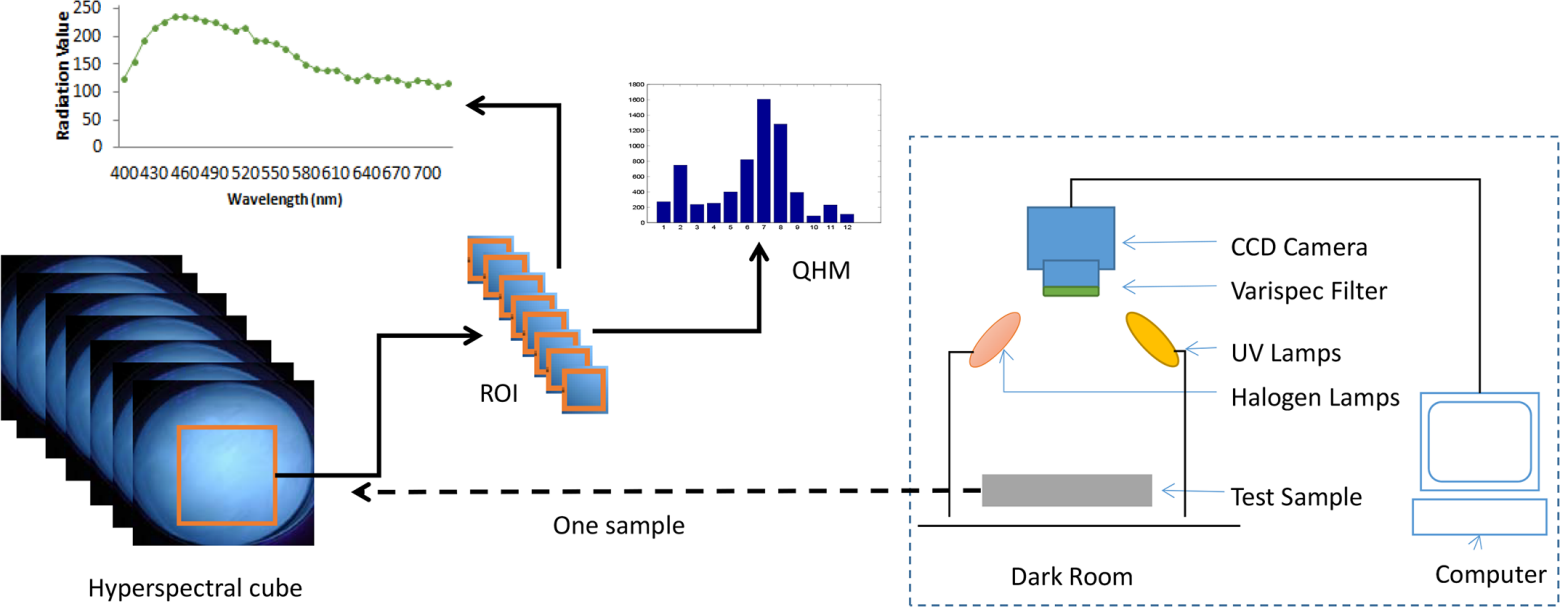
General overview of the hyperspectral imaging system.

**Fig 2:**
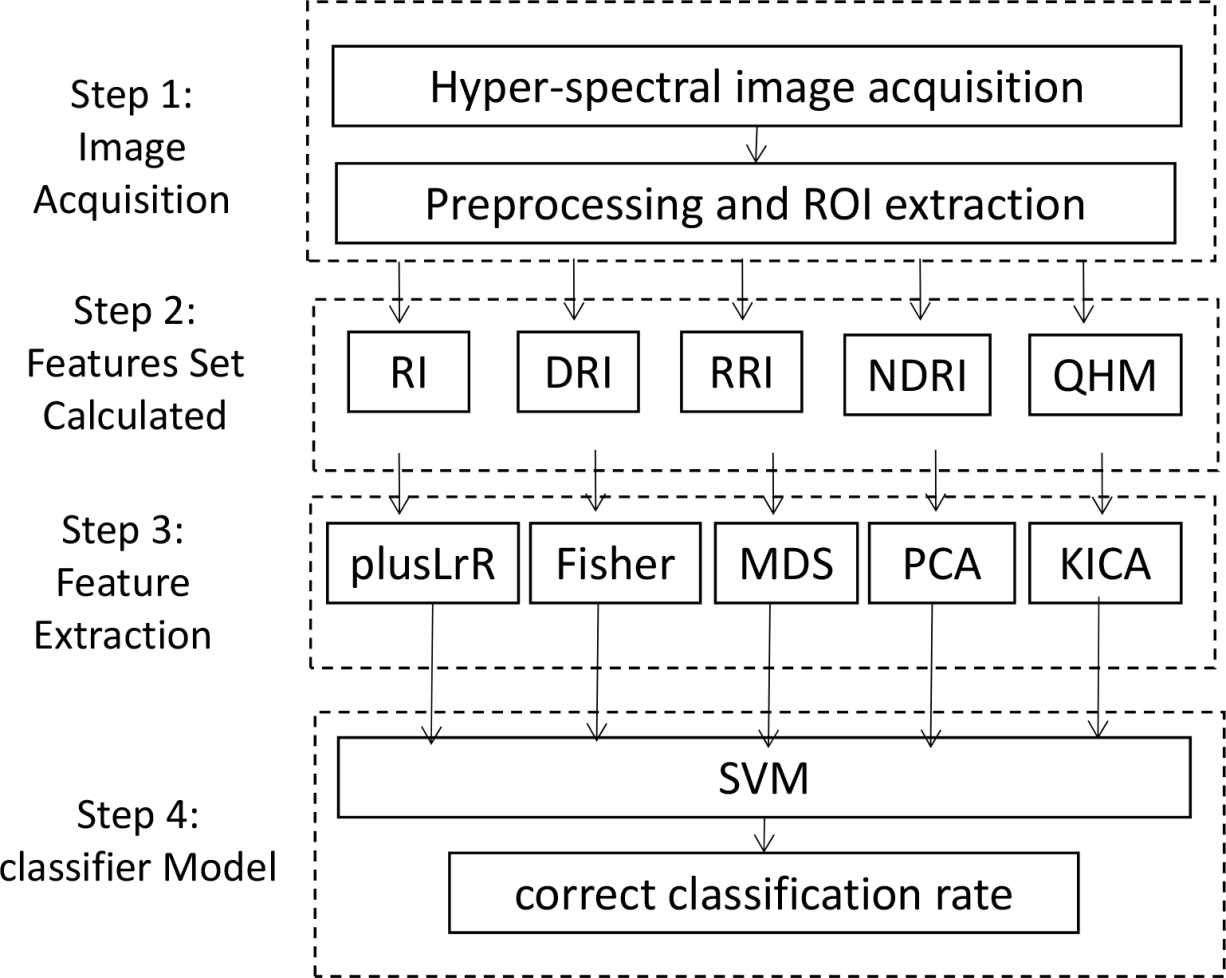
Flow chart and research framework.

In the previous studies [2][3][4][6],single illumination source was used. Especially, some studies were performed just under halogen illumination, and others were performed only under UV illumination. Because UV illumination is utilized for the fluorescence and halogen excitation is for reflectance phenomena. In this study, we utilized both excitations to investigate their contribution to the classification performance. Figs 1 and 2 respectively depict the general overview of the hyperspectral imaging system and the flowchart of the proposed system.

The main steps are as follows. The samples are divided into calibration set and prediction set in a proportion of 4: 1. In the first step, we acquire the hyperspectral images of the four oil varieties within the wavelength region of 400–720nm. In the second step, the reflectance information is extracted from ROI of the hyperspectral images of each sample and used as feature vectors. These feature vectors include individual band radiation index (RI), difference of consecutive spectral band radiation index (DRI), ratio of consecutive spectral band radiation index (RRI) and normalized of DRI (NDRI). Another set of features called quantized histogram matrix (QHM) are extracted from those features by quantizing the histogram of the image. They can be calculated by Eqs 1–5. In the third step, we select significant features based on improved kernel independent component analysis (iKICA). For comparison, some recommended algorithms such as plus L reduce R (plusLrR), Fisher, multidimensional scaling (MDS), principle component analysis(PCA) and independent component analysis (ICA) are also used to identify the most significant wavelengths or features. In the next step, an identification model is established based on support vector machine (SVM) and optimal identification model is selected by comparing the identification performance (correct classification rate, CCR). At last, the result whether the oil samples are qualified or not is achieved by the model.

### Samples

Four varieties of oil products including gasoline, diesel, kerosene and engine oil are from different gas stations or shops in Shandong province, China. Most of them are provided by Shengli Oilfield. There are total 64 samples (ROI) of each variety, and 75% of them are adulterated. All the samples were sent to Shengli Oilfield for oil quality analysis and labeled as qualified oil products and adulterated products. Then, 60 ml of each sample is distributed individually in a glassware with the same size(d = 90mm). And each is then captured individually by the HSI system.

### Hyperspectral imaging system

**Fig 3:**
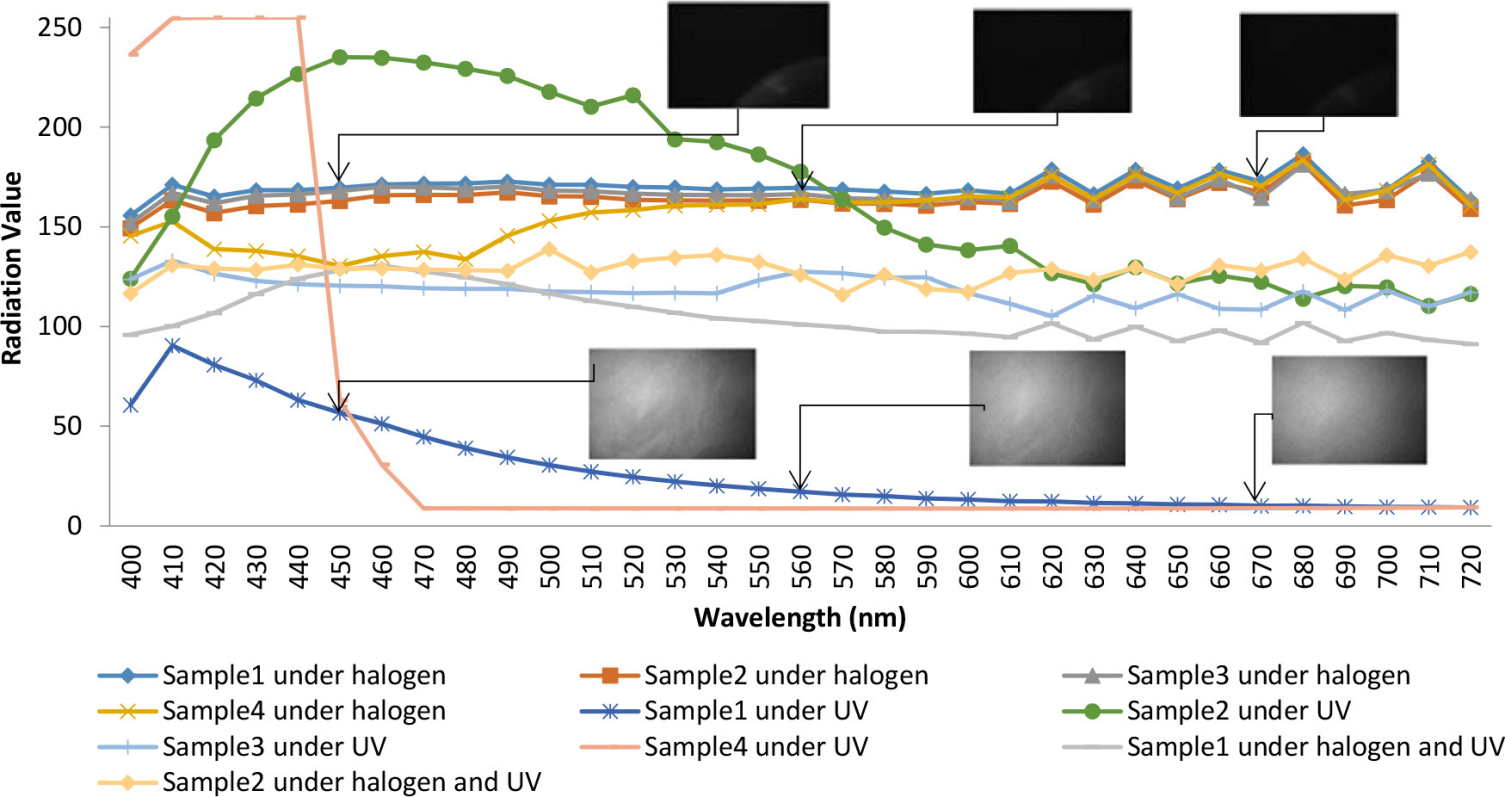
Images and spectraof different Sample.

The image acquisition system consists of a CCD camera with a lens assembly. Hyperspectral image series have been captured under 100W halogen light and UV 365nm LED (LUYOR-3404, USA) illumination sources ranging from 400nm to 720nm with spectral bandwidth 10nm. Size of each image is 1392⤬1020. A raw hyperspectral image (hyperspectral cube) with a dimension of (*x*,*y*,*λ*) which is scanned along the direction of the 33 bands in *λ* dimension is created as the sample. Fig 3 depicts sample images from the hyperspectral image series under halogen and UV illuminations and spectral reflectance curve of different oil samples.

### Preprocessing and ROI extraction

Default camera software uses histogram equalization to acquire images. By adaptively changing exposure time, histogram equalization not only automatically controls over-saturation and under-saturation, but also modifies original pixels’ value. Then, to settle this particular issue, we have to set the value of exposure time and parameter of the camera as a predefined value. Eventually, under saturated and over saturated regions are generated in the hyperspectral image series due to single value of exposure time. Therefore, we multiply a normalization coefficients by reflectance value for all bands. The normalization coefficient is defined as the reciprocal of exposure time. Before feature extraction, the exposure value is normalized by their normalization coefficient. Then we use histogram equalization method to adjust pixel gray values to the range of 0–255.

An area that is considered as the region of interest (ROI) with 174⤬130 pixels is obtained from different locations of each corrected hyperspectral image (each sample) and results in a total of 64 samples of each variety of oil product. Reflectance values of all pixels are obtained by ENVI5.1 software. All features are extracted via Matlab2008a software to establish calibration model for the identification of different oil products.

## Methods

### Radiation index and quantized histogram matrix

Classification performance is closely related to the features extracted from the images. Ideally, the feature vectors should keep the most concise descriptions of the desired function. These feature vectors specify the distinction between qualified oil and adulterated oil. Nevertheless, it is not a straightforward and trivial process to extract meaningful and discriminative features. It requires domain knowledge and underlying physical phenomena. In these hyperspectral images of the oil samples, morphology features do not correlate with difference of oil, and it is not desirable to rely on solely spectral band mean intensity Therefore, useful features should be considered. In this study, we extract features by calculating radiation indexes of consecutive spectral bands and applying histogram quantization method.

Assume the gray value of the pixel located at (*x*,*y*) of the *k*th spectral band is *I*_*k*_(*x*,*y*). The Individual band radiation index (RI)[7] is defined by:

$$RI_{k}=\sum_{x}\sum_{y}I_{k}\left(x,y\right)\;k=1,2,\cdots ,33$$ (1)

Then, we extract the following feature vectors by calculating radiation index of consecutive spectral band.

Difference of consecutive spectral band radiation index (DRI) is calculated by:

$$DRI_{k}=\sum_{x}\sum_{y}\left(I_{k+1}\left(x,y\right)-I_{k}\left(x,y\right)\right)\;k=1,2,\cdots ,32$$ (2)

Ratio of consecutive spectral band radiation index (RRI) is calculated by:

$$RRI_{k}=\sum_{x}\sum_{y}I_{k+1}\left(x,y\right)/I_{k}\left(x,y\right)\;k=1,2,\cdots ,32$$ (3)

Normalized of DRI (NDRI) is calculated by:

$$NDRI_{k}=\sum_{x}\sum_{y}\left(I_{k+1}\left(x,y\right)-I_{k}\left(x,y\right)\right)/\left(I_{k+1}\left(x,y\right)+I_{k}\left(x,y\right)\right)\;k=1,2,\cdots ,32$$ (4)

Here, *x* = 1 *to M*, *y* = 1 *to N*, *M* and *N* correspond to the size of the band image. The feature vectors described in Expressions 1 to 4 reduce the information in a given band to a single value.

**Fig 4:**
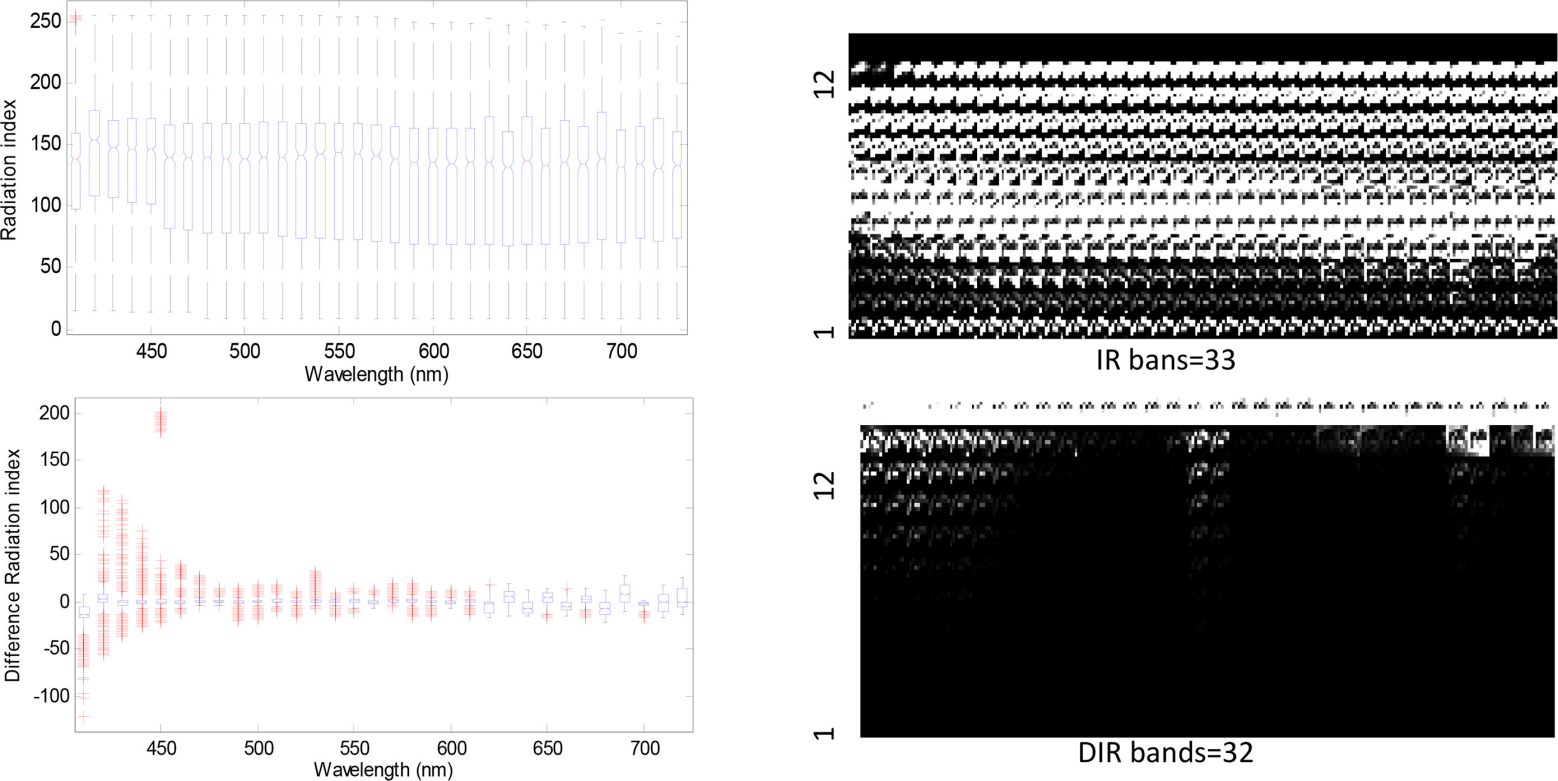
Extracting process of the quantized histogram matrix feature.

However, valuable information may be provided by the frequency of the difference of the intensity values or the frequency of a particular intensity value, which can be extracted when the difference of the intensity values or the histogram of the intensity values for a given spectral band is used. Fig 4 presents the extracting process of the quantized histogram matrix feature. First, the histogram of the spectral band image is computed with a number of bins predefined which not only limits the size of feature vectors but also promotes a reasonable number of pixels falling in each bin. Within the particular bin the total number of pixels is used as the histogram feature. Then we can construct the quantized histogram matrix (QHM) by using all spectral bands depicted in Fig 4. For simplicity, we present the extraction process only for 12 bins.

Here, we only calculat QHM features for RI and DIR. The QHM features can be described as:

$$QHM_{k,n}=\sum_{x}\sum_{y}I_{k,n}\left(x,y\right)\;k=1,2,\cdots ,33\;n=1,2,\cdots ,12$$ (5)

Where *k* denotes index of spectral band, *n* denotes the bin index and 12 is the number of bins that we want to apply. Consequently, *I*_*k*,*n*_(*x*,*y*) is the RI or DIR of the *n*th bin.

### Kernel ICA and its improvement

Independent component analysis (ICA) [8] can be expressed as the problem that a latent random vector *X* can be recovered from observations of *m* unknown linear functions of that vector. The components of *X* are assumed to be independent of each other. And, an observation *Y* is modeled as:

$$Y=AX\;\text{here},\;X=(x_{1},x_{2},\cdots ,x_{m}),\;Y=(y_{1},y_{2},\cdots ,y_{m}).$$ (6)

Where *x* is a latent random vector with independent components, and *A* is a parameter matrix of m⤬m. Given *N* independently, identically distributed observations of *y*, we hope to estimate *A* and thereby to recover the latent vector *x* corresponding to any specific *y* by solving a linear problem.

We can obtain a parametric model which can be estimated via maximum likelihood by specifying distribution for the components *x*_*i*_. With *w* = *A*^−1^ as the parameterization, one can easily obtain a gradient or fixed point algorithm that yields an estimate $\hat{W}$ and provide estimates of the latent components via $\hat{X}=\hat{W}Y$. Hyvärinen et alhave proposed an algorithm named fast fixed-point algorithm for independent component analysis[9].

Unfortunately, it is difficult to approximate and optimize the mutual information based on a finite sample. In this paper, we provide a new solution to the ICA problem based on an entire function space of candidate nonlinearities instead of a single nonlinear function. Especially, the functions are dealed with in a reproducing kernel Hilbert space, which we can use “kernel trick” to search over efficiently. It is the use of the function space that makes it possible to adapt to all kind of sources and makes algorithms more robust to various source distributions depicted as follows.

Bach et al defined a contrast function that can do a rather direct measurement of the dependence of a set of random variables functions from ° to ℝ'[10].

For simplicity, assume *x*_1_ and *x*_2_ are two univariate random variables and *F* is a vector space of functions from ° to ℝ' and *F*-correlation *ρ*_*F*_ is the maximal correlation between the random variables *f*_1_(*x*_1_) and *f*_2_(*x*_2_), where *f*_1_ and *f*_2_ range over *F*:

$$\rho_{F}=\underset{f_{1},f_{2}\in F}{\max}corr(f_{1}(x_{1}),f_{2}(x_{2}))=\underset{f_{1},f_{2}\in F}{\max}\frac{\mathrm{cov}(f_{1}(x_{1}),f_{2}(x_{2}))}{{\left(\mathrm{var}f_{1}(x_{1})\right)}^{1/2}{\left(\mathrm{var}f_{2}(x_{2})\right)}^{1/2}}$$ (7)

It is clear that the *F*-correlation would equal to zero if the variables are independent. Furthermore, the converse is also true if F is big enough.

We use the idea of reproducing kernel Hilbert space (RKHS) to get a computationally manipulable implementation of the *F*-correlation. Let *F* be an RKHS on °, *K*(*x*,*y*) be the associated kernel, and Φ(*x*) = *K*(⋅,*x*) be the feature map, where *K*(⋅,*x*) is a function in *F* for each *x*. Then we have the famous reproducing property.

f(x)=⟨Φ(x),f⟩,∀f∈F,∀x∈ℝ. (8)

This implies:

$$corr(f_{1}(x_{1}),f_{2}(x_{2}))=corr\left(\langle \Phi (x_{1}),f_{1}\rangle ,\langle \Phi (x_{2}),f_{2}\rangle \right).$$ (9)

Consequently, between one-dimensional linear projections of Φ(*x*_1_) and Φ(*x*_2_) the *F*-correlation is the maximal possible correlation that is exactly the definition of the first canonical correlation between Φ(*x*_1_) and Φ(*x*_2_), which suggests that the computation of a canonical correlation can be based on an ICA contrast function in a function space.

The separated independent components (ICs) are unordered using traditional ICA. The first separated ICs may be not important. Therefore, it needs some criteria to sort these ICs. In this paper, we use negentropy as a criterion to measure the nongaussianity of ICs. Then, the IC with maximum negentropy will be separated first. Negentropy is given by

$$N_{g}(Y)=H(Y_{Gauss})-H(Y)$$ (10)

Where, *Y*_*Gauss*_ is a random gauss variable and has the same variance as *Y*, *H*(⋅) is the differential entropy of the random variable.

### Support vector machine

Support vector machine was proposed by Cortes C.& Vapnik V. [11]. SVM has been widely used in many fields [12][13][14], and can solve both linear and nonlinear multivariate calibration problems. Rather than a quadratic programming (QP) problem, a set of linear equations was used to get the support vectors (SV). Here, we utilize support vector machine (SVM) as the classifier for our problem. The radial basis function (RBF) is used as the kernels in consideration of its excellent performance. The SVM algorithm is presented as below:

$$y(x)=\sum_{k=1}^{N}\alpha_{k}K\left(x,x_{k}\right)+b$$ (11)

Where, *α*_*k*_ are Lagrange multipliers, *K*(*x*,*x*_*k*_) is the kernel function, and *b* is the bias value.

The regularization parameter *gam*(*γ*) is used to measure the tradeoff between the training error and model complexity, and parameter *sig*^2^(*σ*^2^) is used to define the non-linear mapping from input space to high dimensional feature space. In this study, the optimal parameter values of (*γ*,*σ*^2^) are calculated by grid search and they are calculated by free LIBSVM toolbox(v2.91) [15] in matlab2008A.

## Results

### Feature construction

Series of hyperspectral images of each oil samples are acquired at two different illumination modes (halogen and UV) within the spectral region of 400-720nm with 33 spectral bands. The spectral information has the characteristic of high dimensionality with redundancy among contiguous wavelengths. Images of the 64 different locations of each sample generate a total of 16896 images of 1392⤬1020 resolution. If the gray value is used as the feature vector directly, the size of it will be too large. Large feature size causes “curse of dimensionality” problem as known to all. As increasing the dimension of the feature vector results in exponential increase in the data size, the size of feature vector should be reduced to a reasonable level. Fewer features have many advantages, such as improving the classifier performance, providing a faster computation and making the underlying mechanism of the problem better understood.

**Fig 5:**
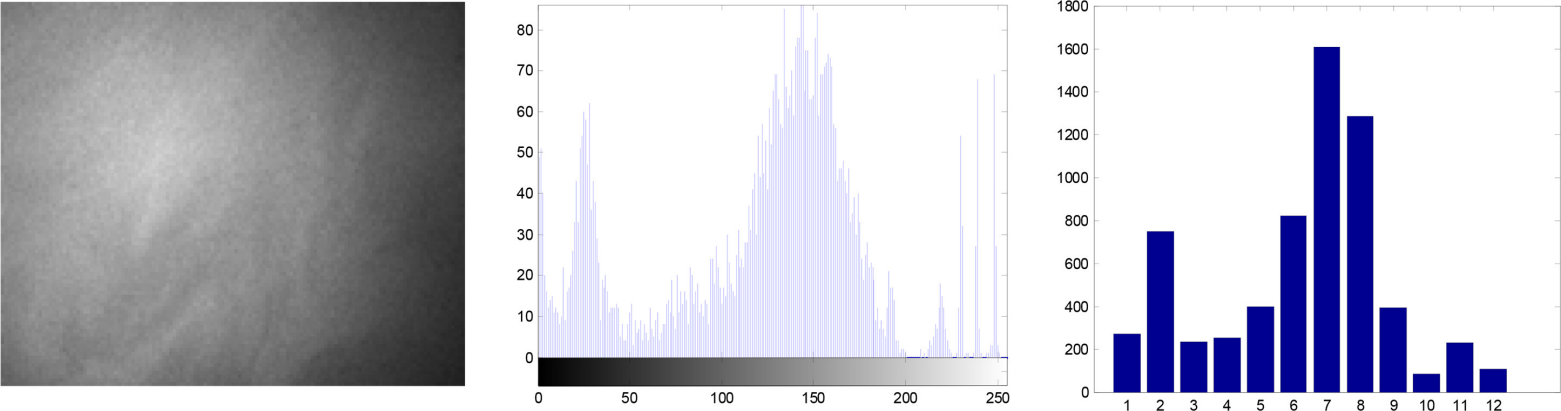
Boxplot of RI, DRI and quantized histogram matrix(QHM) of 12 bins.

By Eqs (1) to (4), feature vectors are extracted with the size of 33 or 32. The other two types of feature sets are extracted according to Eq (3). They are respectively quantized by RI and DRI. The total number of features in the quantized IR is 33(spectral bands)⤬12(quantization bins) = 396. Similarly, we have 384(32⤬12) features for the quantized DRI.The left figure in Fig 5 depicts the boxplot of the IR and DRI under halogen illustration, and their QHM features are shown in the right figure in Fig 5. It is obvious that the DRI data have strong separation ability. As seen the right figure in Fig 5, there exists large number of zero value features in the feature set. These zero feature will be discarded in the first step.

### Features selection

Effective feature selection aims to seek for a subset of features as small as possible to cover the full wavelengths. The subsets of features, as the substitution of the full spectral features, are equal or more efficient because reducing the dimensionality of raw data makes the identification less time-consuming.

**Fig 6:**
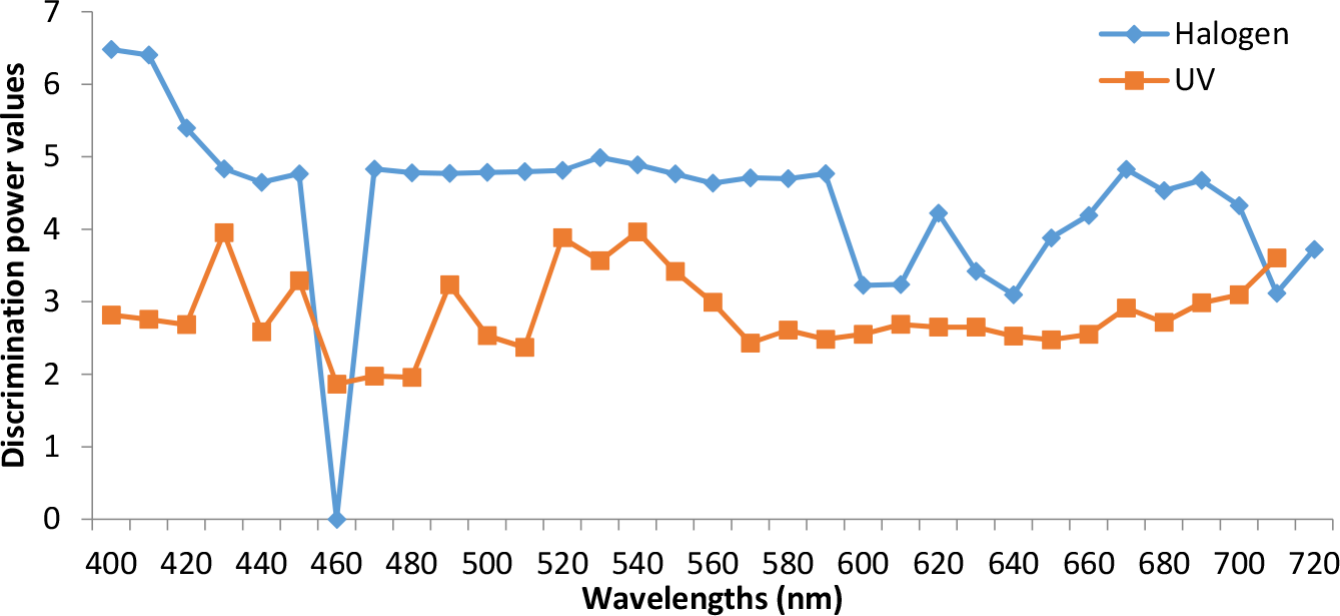
Fisher discrimination power of the DIR.

Plus L reduce R (plusLrR) and Fisher algorithms are used in this paper as features selection methods to identify the most significant wavelengths, which can also be used in the development of the multispectral imaging identification system. With the reduced spectral bands, it will be possible to construct a simple machine vision system for oil detection. Fig 6 illustrates the Fisher discrimination ability values of each band. It is obvious that some bands have stronger discrimination ability, such as 400,410nm under halogen illumination and 430 and 520nm under UV illumination.

Features optimization methods are to seek a nonlinear mapping from all bands wavelength to a feature space, whose size is smaller than that of bands wavelength. We use multidimensional scaling (MDS) and principle component analysis (PCA) for feature detection. Independent component analysis (ICA) has been used to identify the single component spectra in glucose[16]. Kernel independent component analysis (KICA) is a kind of feature detection methods essentially[17]. They can be carried out to identify the most significant feature mapping from feature set. The improved kernel independent component analysis (iICA) proposed in this paper uses negentropy as a criterion to measure the nongaussianity of ICs. The IC with maximum negentropy will be first separated. This will be very useful for classification.

### Classifier results

In this paper we use K-fold cross validation technique to evaluate the generalization performance. In the machine learning community, Wassenaar et al suggest that the recommended value of *K* is usually 5 or 10 [18]. Therefore we set *K* as 5 in this paper. Our data set is randomly divided into five disjoint folds. Four of them are used for training and validation purposes, and the left is used as the test set for our predictive model. The process for each fold is repeated for 5 times to get the average accuracy rate.

To evaluate the classification performance of our method, we compared our method with the original features and reduced features using plusLrR, Fisher, MDS and PCA methods. To achieve a fair comparison, wee unify the size of the feature subsets to 12.

**Fig 7:**
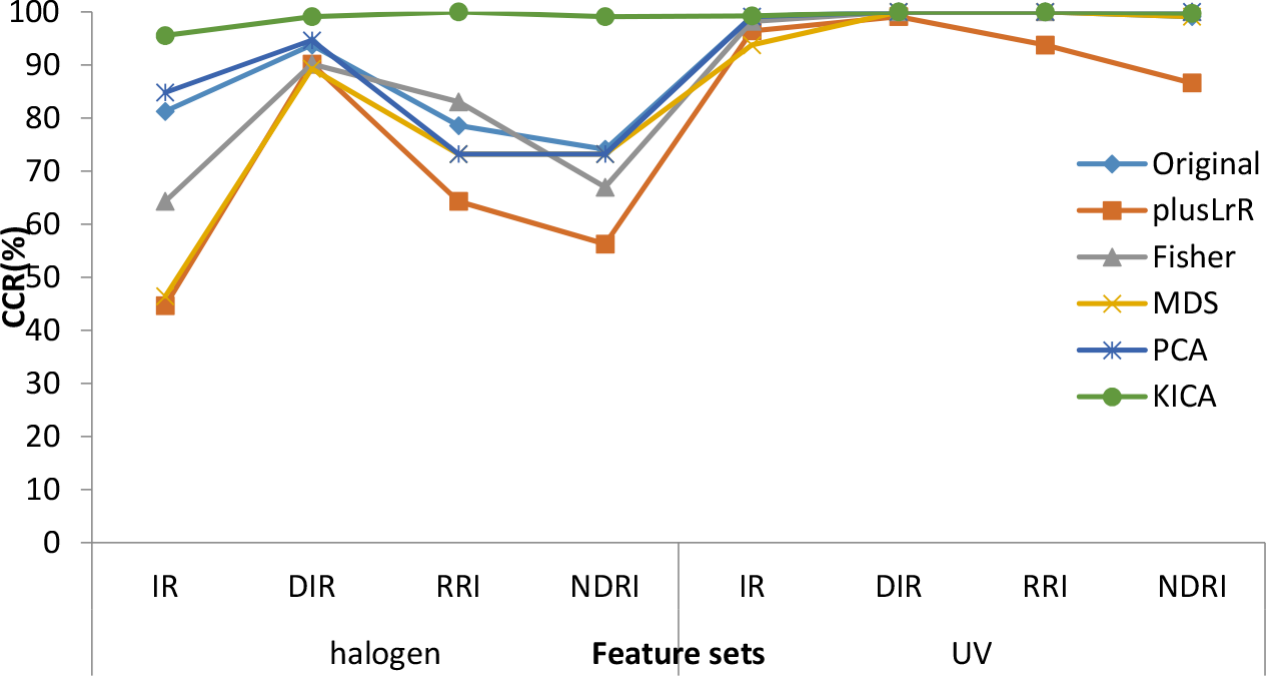
Generation performance of the extracted features with different feature selection methods. Here, there are two kinds of light (halogen and UV).

Table 1 shows overall accuracy rates of several feature sets with various feature selection methods under the halogen and UV illuminations. The best accuracy rates of different feature sets are highlighted in bold. The composite illustration is depicted in Fig 7. As indicated in Table 1 and Fig 7, in most case, iKICA method outperforms the others. Even though Fisher, MDS and PCA can achieve a 100% accuracy rate in two cases (DIR and RRI under UV excitation). iKICA still exhibits the best performance. As can be seen from Table 1, taking operation of consecutive spectral band generally improves the classification performance for both halogen and UV excitations. The DRI is the most perfect.

**Table 1:** Result of the extracted features with different feature selection methods by SVM classifier under halogen and UV excitations.

Illumination source	Feature sets	Org. feature size	Feature selection methods(12features) SVM classifier					
			Original	plusLrR	Fisher	MDS	PCA	iKICA
Halogen	IR	33	81.25	44.64	64.29	46.43	84.82	**95.54**
	DIR	32	93.75	90.18	90.18	89.29	94.64	**99.11**
	RRI	32	78.57	64.29	83.04	73.21	73.21	**100.0**
	NDRI	32	74.11	56.25	66.96	73.21	73.21	**99.11**
UV	IR	33	99.11	96.43	98.21	93.75	99.11	**99.25**
	DIR	32	**100.0**	99.11	**100.0**	**100.0**	**100.0**	**100.0**
	RRI	32	**100.0**	93.75	**100.0**	**100.0**	**100.0**	**100.0**
	NDRI	32	99.11	86.61	100.0	99.11	100.0	**99.75**

Table 2 compares the proposed quantized histogram matrix (QHM) features [19] with texture features proposed by Wang et al. As shown in this table, our proposed QHM features outperform texture features. We compare the result of SVM with that of artificial neural network (ANN). In the ANN, how to determine the optimal number of neurons in the hidden layer is still an open problem. There exist some empirical rules which are widely used [20][21]. Specifically, the Rapid Miner team suggested the number of neurons in the hidden layer could be calculated by[20]:

$$N_{nodes}=\frac{\left(N_{features}+N_{classes}\right)}{2}+1$$ (12)

where, *N*_*nodes*_ is the number of nodes in the hidden layer, *N*_*features*_ is the number of features in input nodes, *N*_*classes*_ is the number of expected classes. In our trials, we used this approach due to the satisfactory results.

**Table 2:** Generation performance of QHM and texture features of DIR by feature selection iKICA and identification by SVM, ANN and PLS under halogen, UV and light fusion.

Illumination source	Feature sets	Org. feature size	Feature selection methods(12features) SVM classifier					
			Original	plusLrR	Fisher	MDS	PCA	iKICA
Halogen	IR	33	81.25	44.64	64.29	46.43	84.82	**95.54**
	DIR	32	93.75	90.18	90.18	89.29	94.64	**99.11**
	RRI	32	78.57	64.29	83.04	73.21	73.21	**100.0**
	NDRI	32	74.11	56.25	66.96	73.21	73.21	**99.11**
UV	IR	33	99.11	96.43	98.21	93.75	99.11	**99.25**
	DIR	32	**100.0**	99.11	**100.0**	**100.0**	**100.0**	**100.0**
	RRI	32	**100.0**	93.75	**100.0**	**100.0**	**100.0**	**100.0**
	NDRI	32	99.11	86.61	100.0	99.11	100.0	**99.75**

In addition, we also compared SVM with linear discriminant analysis (LDA) algorithm.

Table 2 indicates that SVM method outperforms other methods in most cases, and QHM shows higher performance than texture features of DIR. As shown in Table 2, taking both halogen and UV excited at the same time, the CCR will be improved a little. Additionally, the QHM features were more efficient than the band features above, which can be seen from Table 1 and Table 2.

### Method validation

In order to provide evidence for the efficiency of this new method above, here we use another dataset to do the experiment repeatedly. The data set was also collected by hyperspectral camera under two kinds of illuminations (Halogen and UV). There are two kinds of samples, one is crude oil, and the other is crude oil which has been emulsified. Each sample includes 64 hyperspectral data with 33 spectral bands and the resolution is 1392⤬1020. The emulsified oil is the mixture which is composed of crude oil and different percent of emulsification. Here the crude oil indicates the gasoline, and the emulsified oil indicates the adulterationed gasoline.

By the method proposed above, we can get the result as shown in Table 3. As the above conclusion, it is obvious that the proposed method shows the best performance for identifying the crude oil and the oil emulsified. At the same time the DIR provides better feature set than the IR method.

**Table 3:** Generation performance on another set (crude oil and emulsified crude oil).

Illumination	Feature set	Feature selection methods(12features) SVM classifier				
		plusLrR	Fisher	MDS	PCA	iKICA
Halogen	IR	68.21	71.17	91.67	91.67	95.24
	DIR	69.05	95.24	92.53	95.24	97.62
UV	IR	70.24	98.81	97.62	98.81	98.81
	DIR	96.43	100.0	98.81	100.0	100.0

## Conclusion

This paper aims to evaluate the feasibility of identifying the qualified oil and the adulterated oil using HSI with a spectral range of 400-720nm. Hyperspectral image series of 64 oil sample are acquired under both UV and halogen illumination conditions. DIR, RIR and DTIR feature set are extracted based on IR. And then, the most discrimination features QHM are constructed with 12 bins quantization. Besides this, a novel feature selection method has been proposed based on the maximum negentropy of ICs separated by kernel independent component analysis. Compared with plusLrR, Fisher, MDS and PCA methods, our approach achieves a 100.0% accuracy rate under the UV illumination with DIR feature set and KICA feature selection method. UV illumination is superior to halogen. Experimental results demonstrate that SVM outperform ANN in terms of classification accuracy. Robustness of our proposed method is verified by QHM of DIR features under UV excitation with a classification accuracy of 100.0%.

## Supporting Information

S1 File Hyperspectral Imaging Features Dataset. (ZIP)
